# Effectiveness of Robotic Arm-Assisted Total Knee Arthroplasty on Transfusion Rate in Staged Bilateral Surgery

**DOI:** 10.3390/jcm12144570

**Published:** 2023-07-09

**Authors:** Jong Hwa Lee, Ho Jung Jung, Byung Sun Choi, Du Hyun Ro, Joong Il Kim

**Affiliations:** 1 Department of Orthopaedic Surgery, Kangnam Sacred Heart Hospital, Hallym University College of Medicine, 1 Singil-ro, Yeongdeungpo-gu, Seoul 07441, Republic of Korea; bigdawg@hallym.or.kr; 2 Department of Orthopaedic Surgery, Chuncheon Sacred Heart Hospital, Hallym University College of Medicine, 77 Sakju-ro, Chuncheon 24253, Republic of Korea; hodge.jung@gmail.com; 3 Department of Orthopaedic Surgery, Seoul National University Hospital, 101, Daehak-ro, Jongno-gu, Seoul 13620, Republic of Korea; cbsknee@gmail.com (B.S.C.); duhyunro@gmail.com (D.H.R.)

**Keywords:** robotic arm-assisted total knee arthroplasty, transfusion, total knee arthroplasty, staged bilateral total knee arthroplasty, perioperative blood management

## Abstract

The transfusion rate in staged bilateral total knee arthroplasty (TKA) remains high despite the application of blood management techniques. The potential of robotic arm-assisted TKA (R-TKA) in reducing the transfusion rate in staged bilateral surgery has not yet been investigated. Therefore, we aimed to evaluate the effectiveness of R-TKA on transfusion reduction compared with conventional TKA (C-TKA) in staged bilateral surgery. This retrospective study involved two groups of patients who underwent 1-week interval staged bilateral TKA—the C-TKA group and the R-TKA group—using MAKO SmartRobotics (Stryker, Kalamazoo, MI, USA). Each group comprised 53 patients after propensity score matching and was compared in terms of nadir hemoglobin (Hb) level and transfusion rate after each stage of surgery. Both groups showed no significant differences in the propensity-matched variables of age, sex, body mass index, American Society of Anesthesiologists physical status score, and preoperative Hb level. The R-TKA group showed a significantly higher nadir Hb level than the C-TKA group after the second TKA (*p* = 0.002). The transfusion rate was not significantly different between the two groups after the first TKA (*p* = 0.558). However, the R-TKA group showed a significantly lower transfusion rate in the TKA (*p* = 0.030) and overall period (*p* = 0.023) than the C-TKA group. Patients who undergo staged bilateral R-TKA have lower transfusion rate than those who undergo C-TKA. R-TKA may be effective in minimizing unnecessary allogeneic transfusions in staged bilateral surgery.

## 1. Introduction

Total knee arthroplasty (TKA) is associated with substantial blood loss due to bone cuts and soft tissue dissection, often resulting in postoperative anemia and necessitating allogeneic blood transfusion [1,2,3,4]. However, allogeneic blood transfusions can increase risk of periprosthetic joint infection, immune-associated reactions, volume overload, and coagulopathy [5]. These conditions require additional medical care, leading to increased length of hospital stay and higher expenses [6,7].

To reduce transfusion rate, various blood management strategies have been applied, including preoperative hemoglobin (Hb) optimization through iron supplementation before surgery, use of a tourniquet with adequate pressure, preoperative or perioperative administration of tranexamic acid (TXA), and meticulous hemostasis before closing the knee capsule [8,9,10,11,12]. Recently, robot-assisted TKA has been used for accurate surgical planning, resulting in fewer bone cuts, requiring less soft tissue management [13], and leading to reduced blood loss [14]. The haptic technology of the robotic system reduces bleeding by preventing the operator from blindly damaging the posterior soft tissue when cutting through the far cortex of the femur and tibia [15]. Additionally, the three-dimensional array system enables the assessment of knee alignment without reaming the intramedullary (IM) canal, which can cause bone marrow damage and additional bleeding [16].

Previous studies have reported low transfusion rates for unilateral knee arthroplasty, with or without robotic assistance, owing to various perioperative blood management (PBM) strategies. Before the use of PBM, the transfusion rate after TKA was as high as 38% [17]. However, the transfusion rate has reduced significantly (as low as 1.9%) after PBM implementation [18]. This finding suggests that the current PBM strategy is sufficient for reducing the transfusion rate in patients undergoing unilateral TKA. In contrast, the transfusion rate for staged bilateral TKA (SBTKA) remains relatively high. Even with the implementation of PBM strategies, the transfusion rate after SBTKA ranges from 34.7% [19] to up to 96.5% [20]. This highlights the need for a new strategy to lower the transfusion rate after SBTKA. With the advantages of the robot arm-assisted system, the transfusion rate after SBTKA can be reduced by replacing the IM guide with bone pins, less bone resection, and less soft tissue damage. However, no study has compared the transfusion rates between staged bilateral robotic arm-assisted TKA (R-TKA) and conventional TKA (C-TKA).

The primary aim of this study was to evaluate the effectiveness of R-TKA on transfusion reduction compared with C-TKA in staged bilateral surgery. We hypothesized that the transfusion rate after the first TKA is not significantly different between the R-TKA and C-TKA groups but is significantly different after the second TKA.

## 2. Materials and Methods

### 2.1. Patients

**Figure 1:**
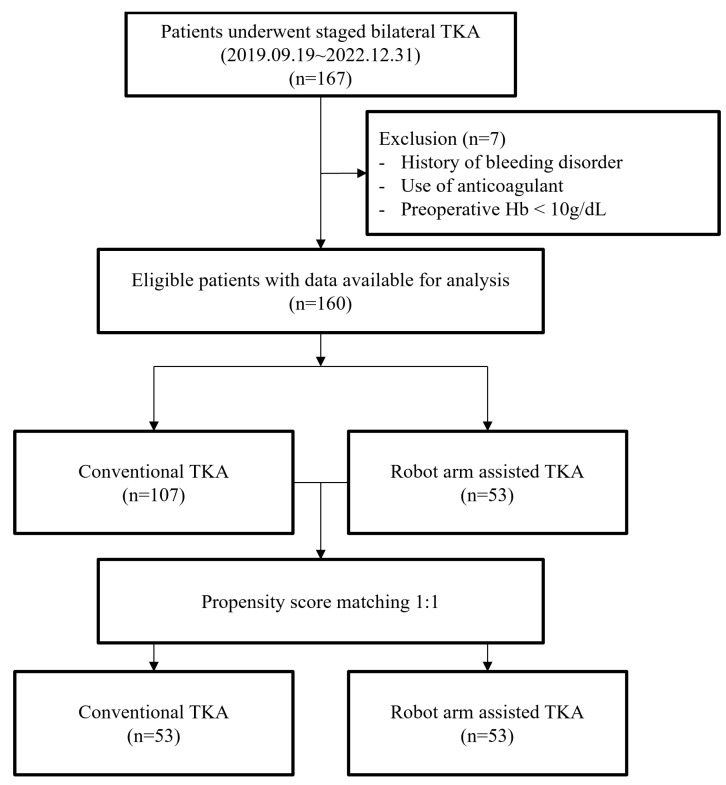
Flow diagram for the patients’ enrollment.

This retrospective study included 167 consecutive patients from a single center who were treated by a single fellowship-trained arthroplasty surgeon. All patients underwent SBTKA 1 week after surgery by either C-TKA or R-TKA between 9 September 2019 and 31 December 2022. The first 112 consecutive patients underwent C-TKA; thereafter, 55 consecutive knees underwent R-TKA after installation of the MAKO Robotic Arm Interactive Orthopedic System (RIO; Stryker, Kalamazoo, MI, USA) in December 2021. Electronic medical records were reviewed to identify patients’ age; body mass index (BMI); American Society of Anesthesiologists physical status (ASA-PS) score; preoperative Hb values; Hb values on postoperative days 0, 1, 2, 4, and 6; and largest decrease in Hb values from the preoperative stage to the postoperative stage (nadir Hb). Hb data after any transfusion were excluded from the statistical analysis because they could be potential outliers. Patients who received a transfusion after their first TKA were not included in the analysis for the second TKA, as the transfusion may have affected their preoperative hemoglobin levels prior to the second surgery. The operation times of the first and second TKAs were recorded. All patients who underwent primary TKA for degenerative or inflammatory arthritis were included in this study. We excluded (1) patients with a history of bleeding disorders with an increased bleeding tendency, (2) those who took anticoagulants for medical conditions, and (3) those who could not meet the preoperative Hb requirements (>10 g/dL) and required a preoperative transfusion. In total, 107 and 53 patients were included in the C-TKA and R-TKA groups, respectively (Figure 1). This study was approved by the institutional review board of our hospital (2023-04-023), and the requirement for informed consent was waived due to the retrospective nature of this study.

### 2.2. Surgical Intervention

Both groups of patients underwent the same surgical protocol, with the only difference being the use of the Robotic Arm Interactive Orthopedic System in the R-TKA group. The medial parapatellar approach was used for all cases, and a tourniquet set at a pressure of 300 mmHg was applied. The tourniquet was inflated just prior to making the incision.

In cases of R-TKA, two pins were inserted into the femur and tibia, 10 cm away from the main skin incision. The femoral and tibial arrays were placed on the pins, and the bone surface was registered. After confirming the patient-specific computed tomography (CT)-based bone model using registered landmarks, the kinematic data were integrated to adjust the CT-based preoperative plan to achieve a balanced knee with functional alignment. Either posterior-stabilizing (PS) prosthesis or cruciate-retaining (CR) prosthesis (Triathlon^®^, Stryker, Kalamazoo, MI, USA) was implanted and final components were cemented in place.

For C-TKA, distal femur resection was performed with IM cutting guide, and proximal tibia resection was performed with extramedullary cutting guide. The femoral entry point was drilled slightly superior to the top of the femoral intercondylar notch. The tibial alignment guide was positioned parallel to the anatomical axis of tibia. Subsequently, it was adjusted to a target slope of 3° in the sagittal plane. The femoral component rotations were determined using a gap balancing technique controlled by the gravity traction method. The PS prosthesis was implanted for all cases and final components were cemented in place.

The tourniquet was deflated after the final fixation of the prosthesis, and the remaining bleeding focus was cauterized after manual compression through gauze packing at the surgical site. A closed suction drain was placed inside the joint, and the capsule was closed in a watertight manner. An intra-articular injection of 1 g of TXA mixed with 50 mL of normal saline was administered inside the capsule, and the solution was left in the joint with the drain clamped. After injection, the knee was moved throughout the range of motion to confirm the watertight closure of the capsule.

### 2.3. Postoperative Management

All drains were removed on postoperative day (POD) 1. From POD 2 to POD 6 of the first surgery, the patients were administered 10 mg rivaroxaban once a day as an anticoagulant to prevent deep vein thrombosis and switched to 100 mg aspirin once a day on POD 2 of the second TKA until POD 6. Range of motion exercises were started on POD 1 after each surgery, and walker ambulation was initiated on POD 2. Transfusion of allogeneic blood was indicated only when the Hb concentration decreased below 7 g/dL or 7–8 g/dL with symptoms of anemia, such as tachycardia and hypotension.

### 2.4. Statistical Analyses

Statistical analysis was performed using the Statistical Package for the Social Sciences software (version 28; IBM, Armonk, NY, USA). To minimize possible confounding factors, patients in both groups underwent 1:1 propensity score matching analysis. The matched variables included age, sex, BMI, ASA-PS score, preoperative Hb level, Kellgren–Lawrence grade (K–L grade), and hip–knee–ankle (HKA) angle. As the number of patients in the C-TKA group (n = 107) was larger than that in the R-TKA group (n = 53), every patient in the R-TKA group was matched to a patient in the C-TKA group. After propensity score adjustment, matched variables were not significantly different between two groups. For continuous data, independent t-tests were used to express results as means and 95% confidence intervals. Pearson’s chi-squared and Fisher’s exact tests were used to compare the percentages of binary data. A *p*-value less than 0.05 was considered statistically significant.

## 3. Results

Both groups showed no significant differences in the propensity-matched variables of age, sex, BMI, ASA-PS score, K–L grade, HKA angle, and preoperative Hb level (Table 1).

The R-TKA group had a significantly higher nadir Hb level than the C-TKA group after the second TKA (R-TKA: 8.55 ± 0.50 g/dL; C-TKA: 8.11 ± 0.86 g/dL; *p* < 0.05). However, it was not significantly different after the first TKA (R-TKA: 9.80 ± 0.99 g/dL; C-TKA: 9.63 ± 1.06 g/dL; *p* > 0.05) (Table 2).

No significant difference in the transfusion rate was noted between the two groups after the first TKA (R-TKA: 1.8%; C-TKA: 3.7%; *p* > 0.05). However, compared with the C-TKA group, the R-TKA group had a significantly lower transfusion rate in the second stage (R-TKA: 7.6%; C-TKA: 23.5%; *p* < 0.05) and overall period (R-TKA: 9.4%; C-TKA: 26.4%; *p* < 0.05) (Table 3).

## 4. Discussion

Our study revealed no significant difference in the transfusion rate after the first TKA between the C-TKA and R-TKA groups. However, the R-TKA group had a significantly lower transfusion rate in the second TKA than the C-TKA group. The overall transfusion rate differed significantly between the two groups. Additionally, the R-TKA group had a significantly higher nadir Hb level than the C-TKA group after the second TKA procedure. To the best of our knowledge, this is the first clinical study to analyze the difference in postoperative transfusion rates after SBTKA with a 1-week interval between R-TKA and C-TKA.

Our study confirmed that robotic arm-assisted TKA procedures resulted in reduced blood loss and eventually reduced transfusion rate after the second TKA. The robotic system links preoperative CT data with intraoperative kinematic data to perform exact bone resection and execute precise implant positioning, requiring fewer bone cuts and soft tissue dissection. Moreover, the haptic boundary prevents the saw blade from cutting through the soft tissue behind the far cortex of the bone [21]. Kayani et al. [22] measured the extent of soft tissue injury based on key anatomical structures to propose a classification system (macroscopic soft tissue injury) and demonstrated that patients undergoing robot-arm-assisted TKA had decreased bone and periarticular soft tissue injuries compared with those undergoing C-TKA. Molloy et al. [23] demonstrated that soft tissue manipulation was associated with the amount of postoperative bleeding, indicating that iatrogenic tissue injury leads to increased perioperative and postoperative bleeding. Robotic arm-assisted TKA prevents iatrogenic tissue injury, leading to reduced bleeding [24,25].

Our study demonstrated that the transfusion rate after the first TKA was not significantly different between the two groups. TKA is a major surgery that is susceptible to substantial bleeding, leading to the need for allogeneic blood transfusion [26]. This led to the implementation of PBM [3,27]. Lee et al. [28] demonstrated that significant decrease in transfusion rate was achieved with oral iron supplement and tourniquet use. Similarly, Morais et al. [29] demonstrated that preoperative Hb optimization through IV iron injection, perioperative tourniquet use, and TXA injection resulted in a zero transfusion rate. This indicates that traditional PBM with additional TXA significantly reduces the transfusion rate. In our study, PBM was performed, using a tourniquet with an adequate pressure of 300 mmHg, intraoperative TXA injection, and meticulous hemostasis, before closing the knee capsule with deflation of the tourniquet. Although preoperative Hb optimization such as intravenous iron supplementation was not performed in our institution, patients with preoperative Hb levels < 10 mg/dL were excluded from our study. With these measures, the transfusion rate after the first TKA was low in both the R-TKA (1.8%) and C-TKA (3.7%) groups and was not significantly different between the two groups. This finding indicates that preoperative Hb optimization, tourniquet use, and TXA injections are sufficient to reduce the transfusion rate in unilateral TKA.

Our study also demonstrated that the transfusion rate after the second TKA differed significantly between the two groups. No study has compared the transfusion rates of SBTKA between the R-TKA and C-TKA groups before our study. In our study, the nadir Hb level after the first TKA was significantly higher than that after the second TKA. Chen et al. [30] demonstrated that Hb level continually decreased until POD 4 and started to recover on POD 5. The ongoing occult blood sequestration from the first TKA site accumulates and affects the second TKA. Recovery of Hb level continued on POD 6 but was still lower than the preoperative Hb level, and additional blood loss inflicted by the second TKA further lowered the Hb level. The preoperative Hb level before the second TKA was lower than that before the first TKA, and the value was closer to our transfusion indication of 8 g/dL, which indicates a higher likelihood of transfusion after the second TKA with even a slight drop in Hb level after the second surgery. All surgeries were performed using the same procedures and PBM with the only difference being the use of robotic system. This indicates that the procedural difference between the R-TKA and C-TKA groups is the primary factor in lowering the transfusion rate. This proves that precise bone cuts, less soft tissue injury, and use of bone pins instead of an IM guide maximize the reduction in unwanted bleeding and transfusion. However, it remains unclear which robot-arm-assisted TKA procedure contributes the most to reducing blood loss. Therefore, further studies are required.

Our study had a few limitations. First, this was a retrospective study, which is subject to a selection bias. To overcome this limitation, propensity score matching was used to match patients with similar demographic characteristics, physical status, and osteoarthritis severity. Second, the decision to perform transfusion was dependent on the surgeon’s preference. In this study, restrictive transfusion thresholds were implemented, and transfusion was not administered until the Hb level reached 7 g/dL. However, in patients who were hemodynamically unstable or presented with anemic symptoms, such as tachycardia, transfusion was performed with a higher Hb level (7–8 g/dL) [3]. Depending on the operator, different decisions may be made regarding whether to observe a patient with an acute onset of anemic symptoms or to transfuse immediately. This may have led to a potential bias. Third, our study was conducted on patients who underwent SBTKA with a 1-week interval, but the results of this study could differ with varying time intervals. However, the optimal interval for SBTKA remains controversial [31,32,33]. Chen et al. [34] demonstrated that a second TKA performed > 90 days and < 270 days after first TKA had fewer complications. However, such a long interval requires re-admission of patients for second TKA surgery, and enduring pain and discomfort of contralateral knee until second surgery could be very difficult for patients. Johnson et al. [35] demonstrated that bilateral TKA staged at 1-week interval was safer than a longer time interval in terms of overall complication rate. In addition, 1-week interval between two surgeries was based on patients’ needs of two surgeries within a single admission and surgeon’s preference. Liu et al. [36] demonstrated in the meta-analyses that simultaneous bilateral TKA showed increased mortality, pulmonary embolism, and deep-vein thrombosis but lower risk of deep infection and respiratory complications compared to staged bilateral TKA. This indicates that both procedures have risks and benefits, and the surgeon’s preference and experiences play huge a role in the decision of optimal timing. Further studies should be conducted to determine whether varying time intervals between bilateral surgeries result in different transfusion rates. Finally, some R-TKA cases with mild to moderate deformities were performed using the CR prosthesis type, whereas all C-TKA cases were performed using the PS prosthesis type. As the CR prosthesis type does not require box preparation, less bleeding can be expected [37]. However, Mähringer-Kunz et al. [38] demonstrated that the transfusion rate was not significantly different between the two groups. Therefore, we assumed that the effect of the difference between the two techniques was negligible.

## 5. Conclusions

Patients who undergo staged bilateral R-TKA have lower transfusion rate than those who undergo C-TKA. Therefore, R-TKA can be an effective strategy for minimizing unnecessary allogeneic transfusions in SBTKA.

## Figures and Tables

**Table 1:** Propensity score-matched data.

Characteristics	C-TKA (n = 53)	R-TKA (n = 53)	*p* Value *
Age, years	70.6 ± 7.5	72.3 ± 5.9	0.187 ^a^
Sex (F:M)	42:11	45:8	0.447 ^b^
BMI (kg/m^2^)	27.2 ± 3.6	27.7 ± 3.4	0.459 ^a^
ASA physical status score (1/2/3)	2/37/14	1/36/16	0.788 ^b^
Preop Hb level	13.4 ± 1.4	13.1 ± 1.2	0.177 ^a^
1st TKA			
HKA angle (°)	9.9 ± 5.3	8.6 ± 5.5	0.203 ^a^
K–L grade (III/IV)	13/40	13/40	1.0 ^b^
2nd TKA			
HKA angle (°)	7.3 ± 5.8	8.0 ± 4.1	0.473 ^a^
K–L grade (III/IV)	22/31	21/32	1.0 ^b^

* Statistically significant *p*-values are shown in bold. C-TKA, conventional total knee arthroplasty; R-TKA, robotic arm-assisted total knee arthroplasty; F:M, female–male; BMI, body mass index; ASA, American Society of Anesthesiologists; HKA, hip–knee–ankle; Hb, hemoglobin; K–L, Kellgren–Lawrence; TKA, total knee arthroplasty. ^a^ *t*-test. ^b^ Pearson’s chi square Test.

**Table 2:** Continuous Outcome Measures.

	C-TKA (n = 53)	R-TKA (n = 53)	*p* Value *
	Mean, std dev	Mean, std dev	
Preop Hb level (g/dL)	13.50 ± 1.44	13.15 ± 1.19	0.177 ^a^
1st TKA POD 0	12.65 ± 1.36	12.96 ± 1.39	0.241 ^a^
1st TKA POD 1	11.22 ± 1.19	11.12 ± 1.32	0.671 ^a^
1st TKA POD 2	10.09 ± 1.12	10.13 ± 1.13	0.863 ^a^
1st TKA POD 4	10.03 ± 1.09	10.18 ± 1.06	0.463 ^a^
1st TKA POD 6	10.04 ± 0.97	10.40 ± 1.02	0.069 ^a^
2nd TKA POD 0	10.32 ± 1.25	10.75 ± 1.20	0.078 ^a^
2nd TKA POD 1	8.89 ± 1.05	9.27 ± 0.85	0.044 ^a^
2nd TKA POD 2	8.47 ± 1.01	8.74 ± 0.61	0.110 ^a^
2nd TKA POD 4	9.13 ± 0.85	9.44 ± 0.88	<0.001
2nd TKA POD 6	9.41 ± 1.12	9.55 ± 0.71	0.487 ^a^
Nadir Hb level after 1st TKA (g/dL)	9.63 ± 1.06	9.80 ± 0.99	0.405 ^a^
Nadir Hb level after 2nd TKA (g/dL)	8.11 ± 0.86	8.55 ± 0.50	0.002 ^a^
1st TKA operation time (min)	90.3 ± 10.4	94.8 ± 14.6	0.068 ^a^
2nd TKA operation time (min)	89.4 ± 18.5	93.8 ± 15.3	0.194 ^a^

* Statistically significant *p*-values are shown in bold. Hb, hemoglobin; C-TKA, conventional total knee arthroplasty; R-TKA, robotic arm-assisted total knee arthroplasty; POD, postoperative day; TKA, total knee arthroplasty. ^a^ *t*-test.

**Table 3:** Binary Outcome Measures.

	C-TKA (n = 53)	R-TKA (n = 53)	*p* Value *
Transfusion rate after 1st TKA (%)	2/53 (3.8%)	1/53 (1.9%)	0.558 ^b^
Transfusion rate after 2nd TKA (%)	12/51 (23.5%)	4/52 (7.7%)	0.030 ^b^
Overall transfusion (%)	14/53 (26.4%)	5/53 (9.4%)	0.023 ^b^

* Statistically significant *p*-values are shown in bold. TKA, total knee arthroplasty; C-TKA, conventional total knee arthroplasty; R-TKA, robotic arm-assisted total knee arthroplasty. ^b^ Pearson’s chi square Test.

## Data Availability

The data presented in this study are available on request from the corresponding author. The data are not publicly available.
